# Assembly and disassembly of stress granules in kidney diseases

**DOI:** 10.1016/j.isci.2025.112578

**Published:** 2025-05-24

**Authors:** Jinchai Zhu, Hua Su

**Affiliations:** 1 Department of Nephrology, Union Hospital, Tongji Medical College Huazhong University of Science and Technology Wuhan, Wuhan 430022, China

**Keywords:** Cellular physiology, Molecular biology

## Abstract

Stress granules (SGs), transient membraneless biomolecular condensates assembling dynamically under stress conditions, mainly comprise untranslated mRNAs and RNA-binding proteins (RBPs) to orchestrate cellular stress adaptation. SGs biogenesis progresses through translation arrest-induced mRNA condensation, liquid-liquid phase separation (LLPS)-mediated assembly, and subsequent maturation, while their disassembly enables translational recovery after stress relief. SGs emerge as promising therapeutic targets through dynamic regulation to counteract stress conditions, yet their pathological dysregulation disrupts cellular homeostasis, positioning these dynamic biomolecular as potential therapeutic targets through modulation of their assembly and disassembly equilibrium. While SGs biogenesis-disassembly mechanisms in renal contexts remain enigmatic, this review systematically examines their interplay with pathological state (e.g., hyperosmolarity, acidosis, and aging) and dual roles in renal pathophysiology — facilitating repair or driving progression in acute kidney injury, kidney cancer, and hereditary nephropathies. Our synthesis of SGs mediated stress adaptation mechanisms highlights critical knowledge gaps in nephrology.

## Graphical abstract

**Figure:**
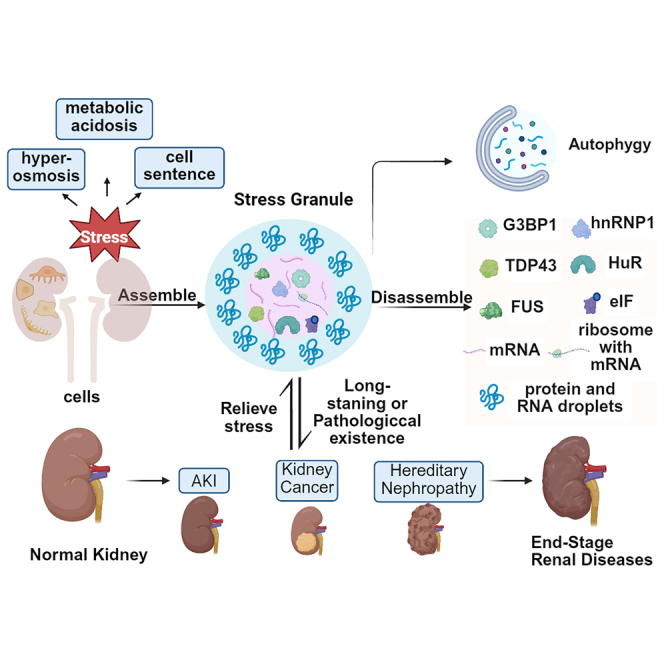

## Introduction

Cells may be exposed to diverse stress stimuli throughout their life cycle, encompassing both environmental challenges such as hyperosmolarity,^1^ nutrient deprivation,^2^ and hypoxia,^3^ causing intrinsic perturbations including reactive oxygen species (ROS) accumulation and cellular pathway dysfunction.^4^ When these stressors threaten cellular homeostasis, organisms have evolved sophisticated defense mechanisms involving transcriptional reprogramming, proteostatic adaptations, and initiation of apoptosis when stress becomes irreversible.^5^ Among these adaptive strategies, dynamic cytoplasmic reorganization through membraneless organelle formation has emerged as a crucial survival mechanism.^6^ Recent studies demonstrate that physicochemical regulatory networks of phase separation precisely control the spatiotemporal dynamics of these condensates via liquid-liquid phase separation (LLPS).^7^^,^^8^

As a core component of the cellular stress response system, stress granules (SGs) are dynamic phase-separated compartments composed of ribonucleoproteins (RNPs) that rapidly assemble during translational inhibition.^9^^,^^10^ These transient condensates execute hierarchical regulation of gene expression by establishing a molecular sorting mechanism — selectively recruiting stalled translation initiation complexes, mRNAs, and RNA-binding proteins (RBPs).^11^^,^^12^ Their rapid assembly kinetics (<15 min post-stress) surpass the temporal framework of transcriptional and proteostatic adaptations, providing immediate survival buffering during acute crises in mammals.^13^^,^^14^ Even in plant cells, SGs can appear within 5 min^15^ Crucially, SGs disassembly upon stress resolution enables rapid resumption of protein synthesis using preserved molecular resources.^16^ The assembly and disassembly kinetics of SGs are regulated by changes in the stress environment as well as post-translational modifications of core proteins such as(G3BP1 [GTPase-activating protein SH3 domain binding protein 1]and TIA-1[T cell intracellular antigen1]).^11^^,^^17^

Emerging research in renal pathophysiology reveals the dual role of SGs. Transient SGs formation protects renal tubular epithelial cells during ischemic preconditioning via protecting m^6^A-methylated mRNAs, sphingosine kinase (SphK) a key enzyme that catalyzes the phosphorylation of sphingosine into sphingosine-1-phosphate (S1P), and promoting cell proliferation and survival.^18^ Whereas merging evidence persistent stress may be conductive to cell survival initially, accumulation exacerbates abnormal SGs and persistent SGs under prolonged hyperosmolarity,^19^^,^^20^ acidosis,^21^ and aging.^22^

This study systematically investigates the morphological characteristics, molecular composition, and assembly-disassembly dynamics of biomolecular condensates under stress conditions.^11^ We think that the interplay between physiological SGs and pathological protein aggregates constitutes a phase transition dysregulation continuum — a core pathomechanism transcending traditional dichotomous classifications. Nevertheless, critical knowledge gaps persist: Current research predominantly focuses on characterizing the physicochemical properties of biomolecular condensates, while largely neglecting to decipher the functional heterogeneity underlying their dynamic evolution in disease contexts.^23^^,^^24^^,^^25^

## What are stress granules and RNP granules?

SGs, transient cytoplasmic foci (100–2000 nm)^26^ observable by light microscopy, represent a specialized subset of RNP granules — a family of non-membrane-bound compartments also and others.^26^^,^^27^ These dynamic condensates assemble under diverse stressors,^2^^,^^28^ through LLPS, serving as hubs for sequestering untranslated mRNAs and RBPs.^11^^,^^29^^,^^30^ This seems to enable RNP granules to function as localized functional units concentrating specific RNAs and proteins.^31^ Consequently, they also serve as cytoplasmic or nuclear microcompartments that establish dynamic sorting stations for mRNAs and associated proteins.^32^ The composition of RNP granules are also influenced by stressors (such as temperature^33^ and intracellular parameters like osmotic pressure, pH, molecular crowding, and metabolite concentration),^33^^,^^34^ which play a role in the biogenesis of RNP granules, enabling cells to adapt and grow under variable environmental conditions.^17^ However, RNP granules are highly concentrated structures that typically contain RNAs, RBPs, and eukaryotic initiation factors (eIFs), making them dynamic related to ATP-dependent processes, static RNA buffering, and dynamic post-transcriptional regulation.^35^ Previous studies have revealed that germ granules and piRNA processing compartments (pi-bodies) share structural similarities. These phase-separated assemblies universally incorporate RNA helicases (e.g., Vasa/DDX4), Tudor-domain scaffolding proteins, and Piwi-family argonaute proteins —components essential for small RNA biogenesis and transposon silencing.^36^^,^^37^^,^^38^^,^^39^ Intriguingly, SGs exhibit partial molecular overlap with processing bodies (P-bodies) and other biomolecular condensates.^27^^,^^40^ This molecular convergence enables functional crosstalk, wherein competition for shared components dynamically regulates mRNA fate decisions under stress conditions through LLPS-mediated partitioning.^41^

**Figure 1:**
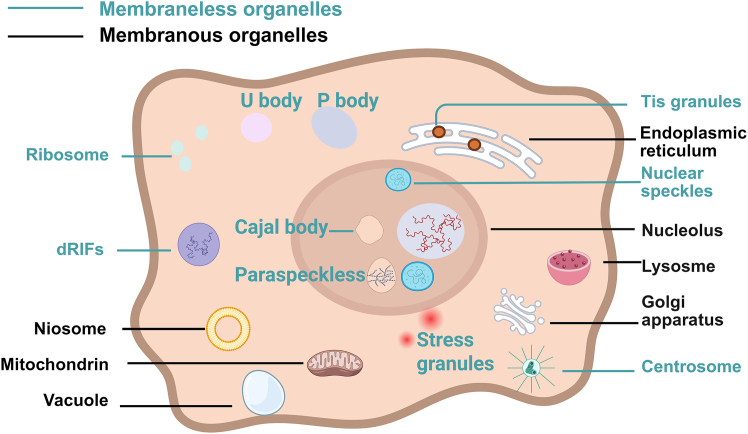
Summary of cellular organelles with and without membranes The following is a summary of the RNA contained therein. Cajal body formed from nascent snRNAs, paraspeckles formed from long (nuclear paraspeckle assembly transcript 1) NEAT1 RNAs, nuclear speckles formed from nascent mRNAs, nucleolus formed from nascent rRNAs, Tis granules formed from endoplasmic reticulum (ER) associated mRNAs and TIS11, P-body formed from untranslated mRNAs associated with decay machinery, U-body formed from snRNPs, dsRNA-induced foci (dRIFs) formed from dsRNA and dsRNA binding proteins, stress granules formed from untranslated RNPs.

Although some of these biomolecular aggregates share common structural features, their functional specialization is manifested through different molecular characteristics. Emerging evidence suggests that RNP granules possess distinct functionalities, with embryonic RNP granules potentially serving as translation factors or facilitating the translation of their target mRNA.^42^^,^^43^ Also, various RNP granules typically harbor distinct RNA populations, such as cytoplasmic populations of SGs and PBs, which predominantly consist of non-translated mRNA molecules as well as the nucleolus that houses rRNA transcripts. The classification of specific types of RNP granules is based on their cell of origin (germ cells and neurons), composition, subcellular localization (nucleus, cytoplasm, and axons), response to stimuli (osmotic pressure and viral infection), dynamic behavior (temporary or permanent), and related functions (sites for mRNA storage/decay).^13^^,^^25^^,^^44^^,^^45^ As shown in Figure 1, the membranous and membraneless organelles are vital components of the cells.

*In vitro* experiments have demonstrated that proteins related to SGs can undergo LLPS and form liquid, hydrogel, and other assemblies with no fixed stoichiometric ratio.^26^^,^^46^ SGs are transient cytoplasmic condensates that arise under diverse stress conditions, briefly serving as hubs for mRNA storage and signaling.^45^^,^^47^^,^^48^^,^^49^ SGs exhibit a biphasic architecture characterized by a stable core enveloped within a dynamically fluid cortical shell because of its constituent components. Furthermore, SGs are compositionally defined by four core elements: translationally stalled mRNAs, small ribosomal subunits (40S) with associated translation initiation factors (eIF3 and eIF4E), RBPs including TIA-1 and FUS, and the scaffolding protein G3BP1 — a multi-domain protein that contains a NTF2-like domain that mediates dimerization and RNA binding, an acidic disordered region that drives LLPS, an RGG motif that promotes multivalent interactions, and phosphorylation sites that regulate stress response.^50^ It coordinates the assembly and disassembly of SGs through dynamic conformational changes. These RBPs orchestrate SGs assembly through multivalent interactions with newly transcribed mRNAs, which act as scaffolds for condensate formation.^51^ Notably, canonical SGs assembly depends on active cytoplasmic mRNA generation, as blocking transcription, splicing, or nuclear export fails to induce their formation. While early hypotheses posited that translationally stalled mRNAs drive SGs aggregation, emerging evidence suggests a more nuanced mechanism.^52^^,^^53^ Both coding and non-coding RNAs localize to SGs, with coding mRNAs potentially linking SGs to inflammatory responses, endoplasmic reticulum (ER) stress, and metabolic regulation.^54^^,^^55^ Non-coding RNAs, though less characterized, may modulate SGs dynamics through structural or regulatory roles. SGs do not directly suppress mRNA translation but instead compartmentalize cellular mRNAs and proteins into distinct spatial pools. Within SGs, mRNAs exhibit heterogeneous mobility: ∼40% remain static, 25% display restricted movement, and 35% diffuse freely.^54^^,^^56^^,^^57^ This compartmentalization may influence mRNA stability, translation timing, or stress-adaptive signaling, though the functional consequences remain an active area of investigation.^55^ Protein composition within SGs is equally dynamic: ∼50% are non-RBPs implicated in stress pathways such as the unfolded protein response (UPR) or recruited via protein-protein interactions.^56^ SGs assembly is tightly linked to translational control mechanisms. Stress-induced inhibition of translation initiation —mediated by perturbations in eIFs— triggers the aggregation of stalled 48S preinitiation complexes. Key components include eIF2α,^58^ eIF3,^59^ eIF4F complex (eIF4A, eIF4E, and eIF4G),^60^ and ribosome subunits 40s.^61^ Intriguingly, non-canonical SGs subtypes may incorporate 60S ribosomal subunits, whereas 80S ribosomes antagonize mRNA recruitment to SGs.^62^^,^^63^ The composition and assembly pathways of SGs are highly context-dependent, reflecting the interplay between specific stressors and cellular adaptation mechanisms. Upon stress resolution, SGs disassemble, releasing mRNAs for either translational reengagement or degradation. This plasticity underscores SGs as critical sensors of cellular homeostasis, integrating stress signals to regulate cell survival and adaptation.^25^

SGs, as membraneless organelles, exhibit remarkably higher dynamism compared to membrane-bound organelles. Unlike organelles enclosed by membranes, SGs undergo rapid assembly and disassembly in response to diverse stimuli, demonstrating exceptional spatiotemporal flexibility.^64^ The formation of SGs requires a global suppression of protein synthesis, most notably the cessation of bulk mRNA translation, coupled with selective upregulation of stress-responsive proteins.^14^ RBPs serve as core structural components of SGs, which coalesce with translationally arrested mRNAs to form SGs core.^65^ And, usually, the free mRNAs that do not contain ribosomes interact with each other first and then interact with G3BP1.^9^ These cores then recruit additional components through LLPS, progressively assembling a dynamic SGs shell.^65^ SGs exert multifaceted functions in cellular stress adaptation. Their primary role involves triaging mRNA populations — selectively preserving transcripts encoding stress-response proteins while suppressing non-essential translation, thereby enabling targeted proteome remodeling to meet environmental challenges.^66^ Beyond translational control, SGs function as molecular sequestration hubs, compartmentalizing key signaling pathways and isolating pro-inflammatory cytokines and apoptotic mediators. This spatial segregation mechanism protects cellular integrity by preventing inadvertent activation of detrimental pathways during stress.^67^ The SG-mRNA interactome exhibits functional plasticity: upon stress resolution, sequestered transcripts are either released for translation reinitiation or targeted to PBs for decay, enabling rapid proteome remodeling aligned with environmental demands.^68^ Emerging evidence reveals constitutive material exchange between SGs and other RNP granules even under non-stressed conditions, mediated by shared RBPs (e.g., DDX6) and dynamic mRNA sorting mechanisms. The basal crosstalk may prime cells for rapid stress adaptation by maintaining a “pre-assembled” interaction network.^68^^,^^69^ While transient SGs formation promotes cellular survival, persistent stress can lead to pathological SGs persistence.^9^ Aberrant, non-resolving SGs accumulate misfolded proteins and dysfunctional RNAs, disrupting proteostasis and serving as nucleation sites for pathogenic aggregates in diseases.^70^ The most prevalent involvement of SGs is observed in the progression of tumors,^71^ neurodegenerative diseases,^72^ and viral infections.^73^ Despite their important roles in cellular stress responses, limited research has been conducted on the functions of SGs in the renal system.

## Stress-mediated translational control

Stress has a profound impact on cellular processes, particularly on translation initiation in eukaryotic cells. Under stress conditions, cells exhibit tRNA fragmentation, disrupted rRNA maturation, epitranscriptomic remodeling, and ribosomal dysfunction (including pausing or collision events), which collectively activate ribosome quality control pathways. These processes drive the integrated stress response (ISR) and culminate in SGs formation.^74^^,^^75^^,^^76^ One of the key factors in this process is the reduction of tRNA_i_^Met^ transfer to the 40s ribosome subunit under various environmental stress conditions, which is crucial for translation initiation. Targeting eukaryotic initiation factor 2α (EIF2α), a component of the eIF2-GTP-tRNA_i_^Met^ ternary complex, decreases the delivery of initiation tRNA_i_^Met^ to the ribosomal subunit in the 40s, resulting in decreased protein synthesis. Translation inhibition can also be initiated by phosphorylation of EIF2α by one or more of the four EIF2α kinases (GCN2, PKR, PERK/PEK, and HRI).^77^^,^^78^ In addition to phosphorylation of EIF2α, disruption of the cap-binding eIF4F complex also leads to translation inhibition and the assemble of SGs.^60^ In fact, the two paths are complementary. SGs can be categorized into two distinct subtypes based on their molecular assembly mechanisms: eIF2α phosphorylation-dependent classical SGs and eIF2α phosphorylation-independent non-classical SGs.^53^ The classical subtype, typically containing the scaffolding protein G3BP1, is triggered by diverse cellular stressors including oxidative stress, ER stress, thermal shock, ultraviolet irradiation, and viral infections. In contrast, the non-classical variant emerges independently of eIF2α phosphorylation status, primarily forming in response to specific chemical stressors such as sodium selenite and hydrogen peroxide exposure. This mechanistic dichotomy highlights the adaptability of SGs formation pathways to different stress stimuli.^79^^,^^80^

eIF2α is responsible for assembling ternary complexes with GTP and initiation tRNA, facilitating the binding of initiation tRNA to small ribosomal subunits. Under stressful conditions, four kinases possess an eIF2α protein kinase domain that leads to phosphorylation of eIF2α Ser-51, resulting in inhibitory GDP-GTP exchange. This prevents interaction with the initiation factor tRNA_i_^Met^ and its delivery to the ribosome, as well as triggering a series of combined stress responses.^81^ The four kinases that phosphorylate eIF2α have distinct roles in the stress response. The general control of non-inhibitory 2 kinase (GCN2) is responsible for monitoring the level of loaded tRNA and responding to nutrient starvation, specifically amino acids.^82^ Protein kinase R(PKR), which monitors the presence of double-stranded RNA and viruses,^83^ is not universally involved in defense against all viruses. The results suggest that another unidentified kinase, eIF2α, triggers an antiviral response against influenza and vaccinia virus.^84^ PKR-like endoplasmic reticulum kinase (PERK), which monitors misfolded proteins and ER stress levels.^85^ Heme regulatory inhibitor kinase (HRI) is responsible for monitoring hemoglobin synthesis and maintaining REDOX levels in erythrocytes.^86^

The eIF4F complex recruits the 43s pre-initiation complex to the 5′ end of mRNA through its cap-binding subunit, eIF4F, thereby facilitating the formation of the 48s translation initiation complex (48s) during eukaryotic translation initiation. This process enhances scanning for the start codon.^87^ It serves as one of the pivotal checkpoints responsible for overseeing the process of mRNA check out. Disruption in any component of eIF4F complex leads to hindered translation initiation and triggers the formation of SGs. Constituent of eIF4F complex are three proteins, namely eIF4A, eIF4E, and eIF4G, each plays distinct roles. Specifically, eIF4A acts as an RNA helicase accountable for unraveling the intricate secondary structure at the 5′ end of mRNA to ensure seamless assembly of the translation initiation complex.^88^ The eIF4E protein functions as a cap-like binding factor that specifically interacts with the 5′ end of mRNA molecules. On the other hand, eIF4G acts as a splicing protein capable of binding both eIF4E and the 3′ end of the mRNA tail. This spatial proximity between the two ends facilitates their circular arrangement, ultimately enhancing translation efficiency.^89^ Thermal shock causes the disintegration of the eIF4F complex, resulting in aberrant eIF4A and eIF4G. Subsequently, eIF4E is recruited into the assembly of stalled RNPs and condensates together with eIF4A and eIF4G.^90^ The interaction between eIF4E and the core protein G3BP1 of SGs leads to translational obstruction.^91^ What’s more, the formation of another EIF3-deficient subtype of SGs is induced by selenite through dephosphorylation of 4EBP1, which relies on the sequestration of eIF4E and the failure of proper formation of the eIF4F complex.^92^ Interestingly, mTOR can phosphorylates EIF4E-binding protein 1(4EBP1) to prevent 4EBP1 from interacting with eIF4E. But under stress conditions, the inactivation of mTOR leads to the inability of eIF4E to participate in normal complex assembly.^60^ However, the association between mTOR and eIF4F complex in SGs remains a subject of debate. In a recent study, it was found that mTORC1 and S6K1, two pivotal regulators of protein synthesis, indirectly promote eIF4A1 ubiquitination, thereby facilitating sustained carcinogenic translation.^93^ Besides the translation inhibition mechanism of 5′tiRNA^Ala^ involves the displacement of eIF4F from the m7GTP cap of mRNA, leading to the induction of SGs formation. Also, the biological activity of 5′tiRNA^Ala^ is specifically observed in the tetramer RG4, which comprises five layers formed by the 5’ -terminal oligoguanine motif within a single tRNA fragment. Destruction of RG4 eliminates the ability of tRNA fragments to trigger the formation of SGs in the body.^74^

## Translation-blocked mRNA condensation

The accumulation of RNAs is the primary prerequisite for the formation of SGs after translation inhibition.^55^^,^^94^ And the simplest and most common form of RNA-RNA interaction is formed by base pairing of complementary single-stranded sequences.^95^ Contrarily, in both plant and mammalian systems, stress-induced mRNAs (such as hsp70) are preferentially translated during the stress adaptation process and selectively excluded from SGs.^96^ The model assumes that SG-related RNA is the mRNA that undergoes translation arrest and is speculated to retain the functional potential for rapid reactivation after stress. However, translation inhibition is not the only criterion for being recruited into SGs, which indicate that the stress response transcripts in SGs are significantly reduced, and the length of the transcripts has a significant positive correlation with the recruitment efficiency of SGs.^97^ This phenomenon has been confirmed in *in vitro* reconstitution experiments, where the elongated RNA length enhances the phase separation process initiated by the SG-starting protein G3BP1^9^ and single-molecule studies have established a direct relationship between mRNA length and the retention time of SGs. Transcriptome analysis of purified SGs cores identified a length-dependent enrichment pattern, where longer transcripts are more likely to be distributed into these biomolecular condensates. However, it cannot be ruled out that the large-molecular-weight RNAs are more prone to precipitate in the extracted RNP granules due to multiple centrifugation steps in the experimental methods,^98^ and the short transcript may be associated with a rapid response.^99^ Other studies have shown that only 185 mRNAs exhibit significant accumulation in SGs, while the integration degree of most cellular transcripts is extremely low.^100^^,^^101^

## Liquid-liquid phase separation mediated condensate assembly

Protein-free and unfolded mRNA autonomous concentration coupled with G3BP1 conformational rearrangement and heterotypic polyvalent interaction may be the general principle of SGs assembly.^102^ The formation of RNP condensates is initiated when RNA-binding nucleating proteins exceed their critical threshold concentration for LLPS, driving spontaneous biomolecular condensation through cooperative multivalent interactions.^103^ The Flory-Huggins polymer physics framework (1940s) is in line with this process.^104^ While an alternative model suggests that SGs formation may initiate through oligomerization of mRNP granules into stable core structures.^14^ The LLPS paradigm remains the predominant mechanistic model for SGs assembly.^105^ While scaffold protein G3BP1-centric models have significantly advanced our understanding of SGs assembly, they may present an oversimplified view of stress-induced biomolecular condensation. G3BP1/G3BP2 has been demonstrated to be the central node of the core SGs network. The three different intrinsically disordered regions (IDRs) of G3BP1 constitute a molecular switch based on self-inhibition, as evidenced by several study.^51^^,^^106^ G3BP is dispensable for the formation of SGs in response to certain stressors.^107^

Crowding-induced LLPS arises from polyvalent weak interactions where supersaturation triggers coacervation – the demixing of dense biomolecular droplets from cytoplasm through entropic volume exclusion.^108^ During this process, condensate maturation involves spontaneous molecular reorganization, establishing dynamic interfaces between phase-separated dense phases and dilute cytoplasmic regions.^109^^,^^110^ Persistent molecular crowding under sustained stress conditions perpetuates LLPS progression, a process facilitated by multivalent interaction networks involving modular protein domains, prion-like regions, and IDRs.^55^^,^^111^^,^^112^

These interactions are governed by diverse physicochemical forces: hydrophobic interactions, hydrogen bonding, and others. As the Src homology 3 (SH3) domain in the Nck protein, interacts with the proline-rich motif (PRMs) in N-wasp to assemble higher-order oligomers.^17^^,^^113^ Some proteins characterized by prion-like domains which refer to a class of protein domains that possess self-propagating abilities and can lead to the misfolding of proteins, this phase separation of Sup35 requires the C-terminal domain, which binds to Sup45 to form the translation termination complex. Sup35 isolated from Sup45 can be co-located not only with Sup45, but also with the SGs marker protein Pub1.^114^ Proteins containing IDRs lack a durable three-dimensional structure, but a common feature of IDRs is that they are composed of low complexity sequence regions (LCRs), such as a repeat sequence of a single amino acid.^115^ According to the length and number of IDRs and the characteristics of IDRs sequence, the phase transition ability is different, which provides the basis for the interaction between polyvalent weakly adhering molecules. IDR-rich PH-Gly repeats in DEAD-box RNA helicase like DDX3/DDX4, cation-π interactions of Arg residues in aromatic rings and possible π-filling interactions promote phase separation. Similarly, mutated aromatic residues in BuGZ and Nephrin intracellular domains reduce their ability to phase separate.^116^^,^^117^ Dipole interactions of sequence side chains rich in Gln, Asn, or Ser residues also provide a driving force for phase separation.^118^ In summary, these proteins are similar and interact through multiple domains or modules. Interestingly, the N-terminal and C-terminal domains of Caprin-1 can promote the assembly of SGs and inhibit the disassembly of SGs by affecting LLPS.^119^

Protein post-translational modifications (PTMs) serve as molecular rheostats fine-tuning LLPS dynamics. PTM alters the charge, structure, hydrophobicity to change the abundance, availability, and function of RBPs in response to stress so that cells can adapt to stress or programmed death through changes in mRNA splicing, translation and localization.^120^ Several types of PTMs may occur in IDRs, including phosphorylation, acetylation, methylation, ubiquitination, O-GlcNAc glycosylation, and polyADP ribosylation. These modifications can cause changes in specific amino acid residues in the IDRs, altering their physicochemical properties and affecting interactions between proteins.^121^^,^^122^^,^^123^^,^^124^^,^^125^ G3BP1 is a key nucleating protein of SGs, acetylation of lysine 376 residues in the (RNA Recognition Motif)RRM domain,^126^ methylation of arginine residues in the (arginine-glycine-glycine domain)RGG domain,^127^ and phosphorylation of Serine 149 in the IDR1 domain^51^ which regulate the interaction of G3BP1 itself and interfere with the formation of SGs.

Environmental and physicochemical parameters critically regulate LLPS landscapes. On the contrary, under certain stress conditions, such as nitrate treatment, the phosphorylation level of G3BP1 is reduced, and the assembly of SGs is also inhibited, but its disassembly is not affected.^128^ External physical factors, concentration, also have an impact on the LLPS of biological macromolecules. Some core proteins of SGs are primarily nuclear proteins that enter the cytoplasm under stress, increasing their local concentrations and contributing to LLPS, which promotes the formation of SGs.^21^ Physical regulators such as temperature, both heat and cold can regulate the behavior of LLPS.^34^^,^^129^ Beyond that, changes in PH also affect the interactions between proteins and RNAs.^130^ This multiscale regulatory network allows cells to dynamically tune biomolecular condensate formation in response to specific stressors, with SGs assembly-disassembly equilibrium dictating cellular adaptation versus pathological transformation.

## Formation of stress granules

Ranging from 100 to 2000 nm, the core feature of their biomolecular condensation lies in the self-assembly of numerous components through weak and dynamic intermolecular forces.^131^ Research indicates that their formation process has a phased characteristic: from the initial formation of the condensation nucleus to accumulation of mature granules, and latest formation of terminal stress-induced condensates.^14^ And it has been confirmed that the size of SGs hardly changes during long-term stress.^132^^,^^133^ This dynamic process reveals the essential law of the progressive assembly of the granules. Notably, the manifestation of typical SGs is stress intensity-dependent - low-intensity stress only induces protein component condensation rather than granular accumulation.^14^ A strictly defined microscopically visible biomolecular condensate needs to reach a specific assembly threshold.^14^

It is worth delving into that the intensity of molecular interactions in biological systems lacks clear boundaries, which leads to fundamental difficulties in determining the identity of components. For instance, some transcripts only have transient binding with SGs proteins.^100^ And how to define the threshold of mRNA residence time on SGs to determine its component status? Moreover, separation techniques such as sequencing and mass spectrometry may miss weakly persistent interactions (as shown in experiment).^101^ Notably, specific core components (such as eIF4A1) exhibit preferential binding characteristics during the assembly cascade reaction, suggesting that the assembly process under severe stress conditions has an inseparable phased feature, ultimately forming large lesions.^102^ These findings collectively confirm that the formation of typical SGs depends on the spatiotemporal coordination of multiple components by the cytoplasmic biological transport network.^16^ The key evidence supporting this theory includes: breakdown of microtubules significantly inhibits granules accumulation, and various microtubule motor proteins and their binding proteins promote particle migration and fusion to form larger condensation foci.^134^^,^^135^ Although actin filaments do not directly bind to granules, the lamellar actin network under stress conditions can guide the transport of nascent particles to the perinuclear region and form abnormal structures.^136^ More strikingly, SGs establish spatial coupling with the ER-lysosome system through specific anchoring factors.^16^^,^^137^ Compared with the ATP-independent condensation process *in vitro*, the intracellular granules assembly strictly depends on the ATP energy supply mechanism, which raises an important scientific question: are there essential differences between the molecular aggregates reconstructed *in vitro* and the SGs formed under physiological conditions? Do these differences imply that the *in vitro* reconstruction model is fundamentally different from the physiological state?

## Renal stress determinants

Stress environments drive cellular adaptation through biomolecular condensates via three interconnected mechanisms. Thermodynamically, phase separation is governed by microenvironmental parameters (temperature, osmotic pressure, ionic strength, pH, molecular crowding) that modulate hydrophobic/electrostatic interactions and solvent entropy to induce liquid-liquid phase transitions.^138^ Kinetically, energy-dependent dynamics (ATP hydrolysis, NAD+/NADH sensing) regulate condensate assembly-disassembly rates through ATPases and post-translational modifiers, maintaining non-equilibrium states.^139^ Functionally, stress-responsive pathways integrate these dynamics—the UPR via PERK-eIF2α coordinates SGs formation with proteostasis, while the ISR activates kinases (PERK, GCN2, PKR, and HRI) under stress to phosphorylate eIF2α (Ser51), suppressing global translation to conserve energy while selectively translating repair genes.^58^^,^^101^ Notably, translational repression releases RNA cross-linking constraints on G3BP1, triggering SGs assembly with composition heterogeneity across stress types and cell lineages.^9^^,^^140^^,^^141^^,^^142^ Renal cells (podocytes and tubular epithelia) exhibit heightened sensitivity due to their high metabolic activity and limited regeneration,^18^^,^^143^^,^^144^^,^^145^ where persistent ISR activation may links SGs dynamics to pathological microenvironments—unresolved stress promotes SGs persistence, exacerbating inflammation and fibrosis. Thus, the synchronization of phase separation kinetics with stress signaling underpins both adaptive resilience and disease progression in specialized tissues.

The ER activates the UPR through three pathways—IRE1-XBP1 (regulating chaperone expression), ATF6 (enhancing lipid synthesis genes), and PERK-eIF2α (inhibiting translation while inducing ATF4-mediated stress genes)—to address misfolded proteins or calcium imbalance. Notably, persistent ERS ultimately triggers CHOP-dependent apoptosis.^146^^,^^147^^,^^148^ Importantly, CX3CL1 has been shown to alleviate podocyte ERS and improve mitochondrial dysfunction through modulation of the GRP78/eIF2α/CHOP pathway.^149^ Mechanistically, IRE1α undergoes LLPS to form ER-stress granule (ER-SG) condensates, whose abnormal aggregation may drive pathological protein deposition.^16^^,^^150^

Given their high mitochondrial content, renal tubular epithelial cells exhibit elevated ROS production, whereas podocytes accumulate oxidative damage due to limited regenerative capacity.^151^ Crucially, ROS promotes SGs assembly through phosphorylation or conformational activation of G3BP1 via LLPS. For instance, arsenite exposure inhibits K63-linked ubiquitination of G3BP1, resulting in excessive SGs aggregation.^152^^,^^153^ In podocytes, NOX4 overactivation combined with impaired Nrf2-Keap1 antioxidant signaling exacerbates nephrin oxidation and proteinuria.^154^ Furthermore, ROS activates NF-κB and TGF-β signaling cascades, amplifying tubular inflammation and fibrosis.^155^

Metabolic dysregulation ERS upregulates SREBP1 and FASN, driving *de novo* lipogenesis and renal lipid accumulation while suppressing AMPK activity.^156^ Concurrently, oxidized low-density lipoprotein (ox-LDL) may stimulates SGs formation in mesangial cells through oxidative stress pathway activation, promoting IL-6 release and accelerating atherosclerosis-associated nephropathy.^157^ Under hyperglycemic conditions, PKCα signaling enhances HuR phosphorylation, thereby increasing the stability of pro-inflammatory and pro-fibrotic factor COX-2 mRNA and exacerbating renal fibrosis.^157^

Inflammation and cytokines inflammatory chemokines such as MCP-1 induce apoptotic and pro-inflammatory phenotypes in renal parenchymal cells (podocytes, tubular epithelial cells) via NF-κB/MAPK pathway activation. Simultaneously, pattern recognition receptors (e.g., TLR4) exacerbate inflammation through recognition of damage-associated molecular patterns (DAMPs).^158^^,^^159^ Specifically, immune complex deposition in lupus nephritis triggers podocyte inflammatory injury, whereas cytokine storms in sepsis-associated acute kidney injury (AKI) directly damage renal tubules.^160^ Notably, maresin-1 demonstrates therapeutic potential by suppressing TNF-α, IL-1β, and IL-6 expression to resolve inflammation.^161^ It is clarified that SGs can alleviate inflammatory responses.^162^ Further research is needed on the relationship between renal immunity and SGs.

Toxin- or drug-induced injury exogenous toxins (e.g., cisplatin, arsenite, and cadmium) exhibit distinct mechanisms of dynamic SGs assembly through organelle targeting. Cisplatin induces mitochondrial ROS bursts and DNA damage, activating the ATR-Chk1 pathway to transiently inhibit apoptosis via SGs formation while compromising long-term repair mechanisms.^161^ Comparatively, arsenite promotes G3BP1-dependent SGs assembly and activates SQSTM1/CALCOCO2-mediated autophagy for aberrant granule clearance.^153^ Cadmium (Cd) activates the PERK-eIF2α-ATF4-CHOP pathway, inducing ER stress-dependent ferroptosis which is attenuated by autophagy inhibitors.^163^ Intriguingly, SGs demonstrate nephroprotective effects against cisplatin- or ischemia/reperfusion (I/R)-induced AKI by regulating m6A-modified genes (e.g., YTHDF1) to enhance tubular cell survival.^164^ While acute Cd exposure disrupts cellular junctions, chronic exposure depletes glutathione reserves, precipitating oxidative cell death.^150^

Mechanical stress, hemodynamic alterations, and local hemodynamic perturbations modulate SGs dynamics through mechanotransduction pathways. The hypoxic renal medulla (pO2 ∼10–20 mmHg) restricts mitochondrial oxidative phosphorylation in tubular cells, stabilizing HIF-1α to upregulate both glycolytic and pro-fibrotic genes, thereby driving chronic hypoxia-induced tubulointerstitial fibrosis.^165^^,^^166^ Glomerular hypertension may activates the mTORC1-DYRK3 axis to regulate SGs assembly and disassembly dynamics, critically influencing podocyte survival and proteinuria development.^167^^,^^168^

## Disassembly of stress granules

High concentrations of condensed matter are reversible as a product of rapid reaction mechanisms, so as to SGs. Early ideas about the relationship between mRNA translation and SGs degradation were quite simple. When mRNAs are titrated out of SGs into translation, the SGs will disassemble. During the disintegration process of SGs, the initial shell is separated first, followed by the core. Previously, Wheeler hypothesized that SGs decomposition occurs through multiple steps in which stagnant mRNA is isolated from SGs, resulting in structural instability of the protein complex. SGs break down into smaller molecular condensates and subsequently break down or clear these smaller focal followed by the gradual diminish of SGs.^14^ This hypothesis is consistent with later experimental data suggesting that RNAs play a central role in regulating the assembly and disassembly of translation polymers and the stability of specific mRNA transcription.^169^ In addition, depletion of SGs core proteins or some proteins interacts with them accelerates SGs disassembly. According to a recent study, hnRNPA2B1 regulates the interaction between G3BP1 and USP10/Caprin-1. Male Hnrnpa2b1 knockout mice accelerates the disassembly of SGs due to a higher ubiquitination level of G3BP1. It is the weaker protein interaction and the ubiquitination-proteasome system reduce the LLPS.^153^ In fact, given the network multivalent model formed by SGs, the simplest explanation is that SGs decomposition is the result of multivalent reduction.^14^

To sum it up, SGs are cleared via ubiquitin-proteasome or autophagy. (1) Chaperone protein: HSP40/70/104 are associated with disassembly of traditional SGs, and AAA+ ATPase valosin-containing protein (VCP)/Cell division cycle protein 48 (CDC48) mediate autophagy.^170^^,^^171^ (2) DNA/RNA helicase: RNA helicases bind ATP and RNA synergistically and exhibit low affinity for RNA after ATP hydrolysis. While the assembly and disassembly of SGs requires ATP participation by default, we can see that RNA helicases regulate RNPs and RNA thereby changing the intermolecular affinity.^172^ Transcriptional purposes determine the dynamics of SGs. Minichromosome maintenance helicase,^173^ RuvB-like helicase^174^ and Bloom’s syndrome protein^175^ all have the function to regulate SGs dynamics. (3) Cytoskeleton: Both microtubule and actin play a role in disassembly of SGs.^48^^,^^136^ (4) Post-translational modification factors.^176^^,^^177^^,^^178^ (5) Proteasomal^179^: ZFAND1 is core influence factor.^180^ In general, the ways SGs is cleared depends on its presence time and external stress. If the condensate caused by stress is stored in a container, the contents must be removed promptly. SGs are temporarily present under most stress conditions is broken down mainly through chaperone protein mediated non-autophagy dependent pathways, thus allowing the recycling of SGs components. SGs and any associated protein aggregation may become one of the pathologic mechanisms. This persistent SGs are cleared by autophagy dependent pathways. If not, these SGs will disrupt the homeostasis of nucleic acids and proteins in the cell and cause pathological damage.^55^

Studies have shown that several human diseases may arise when RNA aggregation exceeds the ability of chaperonins to break down SGs.^181^ In summary, SGs disassemble disordered includes the following several conditions. (1) Severe extreme stimuli, such as severe heat shock stimulation.^182^ (2) Experiencing chronic external stress, such as chronic inflammation or aging.^183^ (3) Protein mutations that promote SGs assembly, such as prion-like domain mutations of hnRNPs.^178^ (4) Protein mutations that promote SGs clearance, such as the mutation of VCP/CDC48.^62^^,^^180^ (5) Inhibition of autophagy, lysosome, and cytoskeleton impairs the assembly of SGs.^62^

## Stress granules: Functions and stage-specific roles

At present, there is no generally accepted specific explanation for the SGs biological phenomenon. Most of the existing explanations are based on morphological or compositional indicators. It has been proposed that SGs have a series of functions, including isolating mRNA and proteins, protecting mRNA and proteins from degradation, promoting enzyme activity by increasing local concentration, minimizing cellular energy consumption, and participating in translation quality control, signal transduction, and cargo delivery.^102^^,^^162^ First, by spatially segregating mRNAs and signaling proteins to modulate transduction cascades, while transiently sequestering molecular cargos for subsequent delivery.^184^ Second, through compartmentalization-mediated stabilization of labile transcripts and stress-sensitive proteins, thereby establishing transient reservoirs that counteract degradation during proteotoxic crises.^185^ Third, via LLPS-driven biomolecular condensation that amplifies enzymatic reaction kinetics, where elevated local substrate concentrations facilitate stress-responsive metabolic rewiring.^32^ Lastly, under stress conditions, overall protein translation is inhibited, but the shutdown of overall translation is achieved through phosphorylation of translation initiation factors. The absence of core factors does not affect the damage to SGs assembly during overall protein synthesis; therefore, the function of SGs is more focused on selective translation, which predominates.^186^ Therefore, more exploration is needed.^97^^,^^101^^,^^187^ In addition, it has been found that the SGs proteins have similar interaction groups before and during stress.^135^ This may indicate that SGs mainly stabilize through enhanced basal interactions, or the interactions of SGs are unstable or refractive for these methods. Besides typical SGs and atypical SGs have different components, and some SGs contain phenotypes that are rare. Therefore, it is concluded that SGs are usually not merely by-products of other cellular changes.^188^

## Stress granule dynamics in diseases: Assembly-disassembly interplay and functional consequences

The formation of SGs is critically dependent on the LLPS capacity of core scaffolding proteins G3BP1/2.^55^ Pathologically, dysregulation of G3BP1 may disrupt SGs homeostasis, as exemplified in Charcot-Marie-Tooth disease (CMT) where mutant proteins abnormally interact with G3BP1, inducing compositional alterations and functional impairment of SGs.^142^ Mechanistically analogous pathways may operate in renal pathologies: aberrant G3BP1 expression or function (induced by oxidative stress or toxin exposure) could compromise SGs assembly fidelity in renal cells, thereby diminishing cellular stress adaptability and exacerbating tubular or glomerular injury.^152^^,^^153^^,^^154^

Notably, chronic SGs persistence observed in neurodegenerative diseases drives pathological protein aggregation and cellular dysfunction.^142^^,^^189^ During renal fibrogenesis, sustained inflammatory and oxidative microenvironments may stabilize SGs, impairing translational reprogramming of pro-fibrotic mRNAs through prolonged sequestration.^155^^,^^189^ This paradigm is similar to silica nanoparticle-induced ERS activating macrophage foam cell formation and atherogenesis, suggesting potential SG-mediated mechanisms in renal fibrosis.^159^^,^^190^

Oxidative stress constitutes a central pathological hub in various nephropathies (diabetic kidney disease,^191^ AKI^192^). Crucially, redox imbalance markers (malondialdehyde [MDA], superoxide dismutase [SOD]) exhibit dynamic correlations with SGs assembly status.^193^ As evidenced by the therapeutic efficacy of Mudan granule combination therapy in diabetic neuropathy through SOD activity restoration, oxidative stress likely modulates SGs assembly kinetics/stability in renal diseases via phosphorylation-dependent regulation of G3BP1’s RNA-binding capacity.^193^^,^^194^ Mutant proteins could hijack G3BP1 to generate compositionally aberrant SGs.^142^ Similarly, renal disease-associated proteins may sequester core SGs components, preventing physiological disassembly.^164^

## Formation of SGs in kidney hyperosmosis state

In pathological states, osmotic stress has been observed previously in various organs and also in cultured cell lines. Osmotic stress, particularly hyperosmotic stress, affects them by altering membrane ion channels, transporter activity, water outflow, and cytoskeleton remodeling. These changes lead to an increase in intracellular ion strength, prompting cells to adopt adaptive mechanisms to maintain normal function. One key adaptive mechanism involves the body’s active uptake of neutral incompatible osmoregulatory substances from the extracellular medium.^195^^,^^196^ This process is mainly driven by the expression of specific transporters triggered by the transcription factor NFAT5(nuclear factor of activated T-cells 5)/TonEBP (tonicity-responsive enhancer-binding protein). The accumulation of these substances helps to weaken the intracellular ionic strength and restore cell volume without increasing the ionic strength within the cell. This mechanism is crucial for normal kidney function, as evidenced by the atrophy of the kidney medulla observed in NFAT5-deficient mice.^197^ In addition to the accumulation of osmoregulatory substances, there is a balance mechanism between SGs and compatible osmotic regulatory substances induced by hyper osmosis. After several hours of continuous hypertonic state, compatible osmotic regulatory substances slowly accumulate to a certain amount in the cytoplasm through specific transporters, thereby reducing the crowding and ionic strength of macromolecules, SGs will also disassemble.^19^^,^^20^ What’s more interesting, during hypertonic stress, SGs are fairly evenly distributed in the cytoplasm, unlike arsenite induced SGs, and their size and localization are likely to part depend on the presence of microtubule(MT).^198^ Surprisingly, when renal epithelial cells set in long-term exposure to high salt, DNA breaks remained even after the accumulation of compatible osmoregulatory substances.^199^ Moreover, the response of cells to osmotic stress varies depending on its severity. In Madin-Darby canine kidney cells, mild to moderate osmotic stress (400 mosmol/l) caused cell-cycle arrest and did not induce eIF2α phosphorylation, it is a response that promotes cell survival. While severe osmotic stress induced apoptosis in a manner dependent on eIF2α phosphorylation.^200^ Also, hypertonic solution inhibits the translation of most cellular proteins, but induces transcription of genes encoding osmolyte transporters and heat shock proteins(such as HSP70), which may be encoded by TonEBP.^201^ Interestingly, hnRNPA1 accumulates in SGs assembled due to eIF2α phosphorylation in the cytoplasm. However, non-eIF2α phosphorylated cytoplasmic SGs are different. We come to a conclusion that phosphorylation of the eIF2α weakens the ribosome entry site within hnRNPA1, thereby continuously inhibiting the translation of mRNA encoding anti-apoptotic proteins. This reduction in protein synthesis exacerbates apoptosis.^20^ In summary, SGs play an important role in the resistance and adaptation of renal hypertonic cells, but the persistence of SGs is not conducive to the normal life activities of the body. Especially when the renal medulla is in a chronic hyperosmotic physiological state such as during diuretic, antidiuretic processes or hypernatremia.

## Formation of SGs in kidney metabolic acidosis state

In metabolic acidosis, the blood pH and bicarbonate concentration decrease, prompting the kidney to adapt by enhancing its glutamine extraction. This adaptation involves increasing the expression of genes coding for key enzymes in glutamine catabolism and various ion transporters. Consequently, a substance known as SGs is produced.^21^^,^^202^^,^^203^^,^^204^ Prior research has shown that renal ammoniation, a process crucial for acid-base balance, mainly occurs in the proximal convoluted tubules of the kidney. During acidosis, a specific enzyme involved in glutamine metabolism is selectively upregulated in this segment of the nephron.^21^ The renal tubule epithelial cells, which are typically static, have the ability to restore amine circulation in response to environmental stimuli. Polyamines play a role in facilitating communication between proliferating cells by promoting the dynamic termination of microtubules around the cells, while SGs help coordinate the cellular response to oxidative stress.^205^ The pH-response element (pH-RE) is a cellular component that enables cells to adapt to changes in the body’s pH levels. It has the ability to interact with various RBPs, including zeta-crystallin, AU-factor 1 (AUF1), Hu-antigen R (HuR), and TIA1. When metabolic acidosis occurs, it can activate signal transduction pathways, leading to increased expression of pH-responsive proteins or covalent modifications.^21^ HuR, a member of the embryonic lethal vision abnormality (ELAV-like) RBP family, is crucial for the formation of SGs.^206^ There is also evidence indicating that HuR can translocate from the nucleus to the cytoplasm and form SGs.^21^ Moreover, the presence of Zeta-Crystal in renal SGs further strengthens our conviction that SGs play a crucial role in regulating renal chronic acidosis.^130^ In summary, during metabolic acidosis, the kidney adapts by altering glutamine metabolism and ion transport, leading to the production of SGs. These SGs, along with pH-responsive proteins like HuR and Zeta-Crystal, play a crucial role in helping the kidney adapt to changes in blood pH and maintain acid-base balance.

## Formation of SGs in cell sentence state

Aging is a complex process that involves multiple changes at the cellular level, including impairments to cell homeostasis.^22^^,^^189^^,^^207^ In the context of kidney aging, these changes can be particularly significant. One area of interest is the biogenesis of SGs, which are dynamic assemblies of mRNA and proteins that form in response to various cellular stress. SGs core nuclear proteins (G3BP1, TIA-1/TIAR, HuR), transcription factor Sp1 and translation initiation factor eIF2α are all key factors that prevent the normal formation of SGs in senescent cells. As cells age, these targets may change, either individually or in combination. It also leads to smaller and more abnormal SGs formation and reduces eIF4G formation,^22^ which could provide a new biomarker to assess cell senescence. However, it is not clear whether this is a universal phenomenon across all cells. Moreover, little is known about the underlying mechanisms. Aging leads to downregulation of nuclear transport factors (e.g., importinα1, CAS and RanBP1) in kidney, leading to dysfunction of karyoplasmic transport, followed by protein accumulation and aggregation in the cytoplasm.^208^ Importin α (KPNA) has been shown to be involved in SGs assembly, so its downregulation may contribute to the impaired biogenesis of SGs observed in senescent cells.^209^ Besides, RBPs such as G3BP1 undergo phase separation in the assembly of the central channel of the nuclear pore, while poly-dipeptide repeats (DPRs) interact directly with RBPs in aging-related disease states, which may affect the correct assembly of SGs.^210^ During the aging process of kidneys, abnormal mTORC1 signaling disrupts the dynamic balance of SGs through dual pathways: On one hand, its phosphorylation of DYRK3 kinase (a key factor inhibiting SGs disassembly) leads to the persistent abnormality of SGs and promotes pathological aggregation of proteins such as G3BP1.^211^^,^^212^ On the other hand, mTORC1 inhibits autophagy initiation by phosphorylating ULK1, blocking the lysosomal degradation of SGs components by autophagy receptors (such as NDP52), forming a “mTORC1-autophagy-SGs” malignant triangle.^153^ In aging cells, mitochondrial ROS bursts and SASP factors (IL-6/TGF-β) further activate mTORC1, forming a positive feedback loop, exacerbating SGs accumulation and releasing pro-inflammatory factors such as HMGB1, and driving TLR4/NF-κB-mediated inflammation and fibrosis.^213^ Target intervention strategies (such as rapamycin inhibiting mTORC1 and SMER28 activating selective autophagy) can restore DYRK3 activity and clear pathological SGs, breaking the vicious cycle of energy metabolism imbalance and oxidative stress.^212^^,^^214^ We can conclude that the assembly of SGs is affected by cytoplasmic transport during renal aging and multiple mechanisms may be involved in the impaired SGs biogenesis observed in senescent kidney cells. Future research is needed to further elucidate these mechanisms and to determine whether these changes are a universal phenomenon across all cells.

## Study of SGs in AKI

The occurrence of AKI is a critical clinical condition associated with significant morbidity and mortality on a global scale. AKI can be caused by various factors, including renal ischemia reperfusion (I/R), sepsis, and exposure to nephrotoxic substances.^144^^,^^145^ The hypoperfusion resulting from ischemia can give rise to hypoxia, while the burden imposed by high flow rates and fluid resuscitation therapy can induce sodium retention, thereby establishing a detrimental cycle.^215^ Under various stress conditions, renal tubule cells often undergo proliferation and apoptosis, accompanied by varying degrees of renal injury. There are several mechanisms through which renal tubules can withstand these injuries. One of these mechanisms is the phosphorylation of eIF2α, which triggers a comprehensive stress response that halts global protein translation and suppresses the formation of SGs. Additionally, in tissues experiencing ischemic reperfusion, toxic damage, or radiation exposure, levels of circulating tRNA derivatives rapidly increase as a result of conformational changes in tRNA structure prior to tissue rupture. These elevated levels may potentially serve as an early marker for tissue damage.^216^ tRNA-derived stress-induced fragments (tiRNAs) inhibited translations, and tiRNA-interacting protein YB-1 (Y-box binding protein 1) helped angiogenin (ANG) facilitate tRNA cleavage and stress-induced translation inhibition. Interestingly, YB-1 was also found to be localized to SGs in the pathological state of renal osmotic stress.^197^ Further investigation revealed that tiRNAs directly or indirectly bind to eIF4G, eIF4A, or eIF4G/A complexes to inhibit translation.^217^^,^^218^ As previously reported, the interaction between initiation factors and the 5′ end of the mRNA is more stable. Transfection of 5′ - rather than 3′ - ribonucleic acid causes conformational changes when eIF4G or eIF4A is replaced by tiRNAs from mRNA, leading to affinity changes and translation inhibition and facilitating the assembly of SGs. tiRNAs can also displace eIF4F from the m7GTP cap, which significantly inhibits protein synthesis.^218^^,^^219^

Gladys Lee et al. reported that L-carnitine reduces renal tubule cell apoptosis through ROS and ER stress regulation.^220^ Another study has also shown that the expression of ER stress-induced ANG depends on the activity of the transcription factor X-box binding protein 1 (XBP1) in mouse kidney and human renal epithelial cells, which promotes cell adaptation to ER stress by inducing SGs and inhibiting translation.^221^ ROS can increase abnormally under hypoxia and reperfusion conditions and lead to modifications of lipids (especially arachidonic acid), proteins (including nitration, chlorination, and bromination, which are all associated with inflammation), and deoxyribonucleic acid (DNA). SGs can regulate the level of ROS by affecting the expression of ROS-related mRNA and related protein.^222^ G3BP1 and USP10 (ubiquitin carboxyl-terminal hydrolase 10) play an important role in the antioxidant effects of SGs. Studies have shown that USP10 alone does not have the function of resisting oxidative damage caused by hydrogen peroxide, but only after the formation of SGs.^152^ In the non-stress state, USP10 expression is inhibited by G3BP1. When the SGs are formed, the inhibition of G3BP1 on USP10 is weakened, and then ataxia telangiectasia mutated (ATM) helps USP10 with the antioxidant function, which confirms that the SGs are the key oxidant reducing agent under stress conditions.^152^ Both glycolytic inhibitors (2-deoxy-*d*-glucose and 3-(3-pyridinyl)-1-(4-pyridinyl)-2-propen-1-one) and mitochondrial respiratory inhibitors (azide and CCCP) induce SGs formation in mammalian tubular cells, and G3BP1 knockdown cells formed fewer SGs and had higher cell death levels than cells transfected with empty plasmids after cisplatin or azide treatment. G3BP1 is the core protein of SGs, which is the basis of homeostasis under stress. Reintroduction of G3BP1 reversed the level of tubular cell death.^51^ Under *in vivo* and *in vitro* cadmium stimulation, renal ATF6(activating transcription factor 6)and IRE1(Immunoglobulin-Regulated Enhancer 1) pathways synergistically induce apoptosis through CHOP(C/EBP Homologous Protein) induction, XBP1 activation and JNK phosphorylation, while PERK-EIF 2α pathway promotes SGs assembly and anti-apoptotic process.^150^ In addition, mRNA associated with tubular cell survival stored in SGs and disassemble to restore translation after stress recovery also reduces tubular cell death and ischemic kidney injury.^18^ YTHDF1(YTH N6-methyladenosine RNA binding protein 1) attenuate kidney damage under stress by wrapping m6A-methylated mRNAs in SGs.^164^ And the O-GlcNAc modification of YTHDF1 regulates the assembly, stability, and breakdown of SGs, enabling the translation restoration then better recovery from stress.^223^ However, a recent study on the role of GSTM3P1 (mouse homology is gstm2-ps1) in renal ischemia has been reported, in which gstm2-ps1 is significantly upregulated in the proximal tubules in the early stages of ischemic AKI. It exacerbates ischemic AKI by interacting with mir-668, a kidney-protective microRNA, and promoting its degradation. This transient induction of gstm2-ps1 depends on G3BP1, a key component of the SGs,^224^ but the formation of SGs does not contribute to the renal response against ischemia and hypoxia at this stage. This observation can potentially be attributed to the distinct impacts of stress intensity and duration on renal function.

## Study of SGs in kidney cancer

The origin of kidney cancer particularly within the tubular epithelium of the kidney, underscores its global prevalence^225^ Cellular stress is an inherent characteristic of cancer and a crucial driver of tumorigenesis. Recent research has demonstrated that non-membrane organelles known as SGs promote tumor development in response to cellular stress. SGs play a vital role in the adaptive response to cancer stimulation, with slightly released tumor-related cellular stress factors acting as prohormone stimulators that regulate SGs formation, thereby influencing various cellular processes and ultimately contributing to the survival of cancer cells.^226^^,^^227^ SGs modulate the biological behavior of tumors by participating in a diverse array of tumor-related signaling pathways. Verrucarin A (VA), a small molecule derived from the fungal plant pathogen *Myrothecium verrucaria*, has been identified as a selective inhibitor of cell proliferation in clear cell renal cell carcinoma (ccRCC). In VHL^+/+^ cells, VA exerts its inhibitory effect on translation immediately after initiation, leading to an upregulation of phosphorylated EIF2α through exogenous signaling pathways. This is accompanied by an increase in monomeric 80S ribosomal content and a decrease in polysome content, ultimately resulting in the assembly of SGs. Simultaneously, SGs capture the signaling molecules that initiate the apoptosis signaling cascade in RCC cells to attenuate cancer cell apoptosis.^228^ The reason for this could be attributed to the fact that VHL plays a crucial role in the successful assembly of SGs in renal cell carcinoma cells, and a majority of renal cell carcinomas exhibit functional deletion mutations in the VHL gene.^229^ The possibility of utilizing translation initiation inhibitors to target VHL^−/−^ cells in the treatment of RCC arises.^228^ Actually, the isolation of these signaling molecules in SGs effectively eliminates stress-induced apoptotic signals.^230^ Previous studies have shown that sorafenib effectively inhibits the growth of RCC.^231^ Sorafenib induces eIF2α phosphorylation by GCN2, which leads to the formation of well-colocalized SGs (HuR, TIA-1 and G3BP1 markers) and resistance to RCC. When the expression of GCN2 was inhibited, the formation of SGs was decreased and apoptosis was increased. It may be that the anti-apoptotic gene cyclooxygenase 2 (cox2) is recruited into SGs.^231^ Tuberous sclerosis (TSC) is caused by mutations in the tumor suppressor genes TSC1 or TSC2. The TSC2 protein binds to high-density lipoprotein binding protein (HDLBP). HDLBP is the core protein of a SGs, TSC2 physically interacts with HDLBP localized to SGs. Nodular sclerosis accumulation in the kidney leads to renal angiomyolipoma. In the corresponding lysates, G3BP1 and Caprin1 are increased compared to normal kidney, demonstrating the adverse effect of SGs in TSC2-deficient cells on nodular sclerosis. Targeting SGs can reduce tuberous sclerosis and its cumulative effect on the kidney.^232^^,^^233^ G3BP1 has been found to be involved in the regulation of multiple cellular functions, and it promotes RCC tumor progression, metastasis and induce EMT transition through the IL-6/G3BP1/STAT3 signaling axis in RCC.^234^ In addition, the interaction between YB-1 and G3BP1 promotes RCC metastasis.^235^ Except these, modulation of the PLAG1/MEG8/miR-495-3p/G3BP1 network contributes to the diagnosis and treatment of RCC.^236^ And we discover that targeting Circ_0000798 to regular miR-589-5p/G3BP1 axis can inhibit the growth and motility of renal cancer cells.^237^ These evidences of the role of G3BP1 in RCC may provide some ideas for the exploration of SGs in the occurrence and development of renal cancer.

## Study of SGs in hereditary kidney diseases

**Figure 2:**
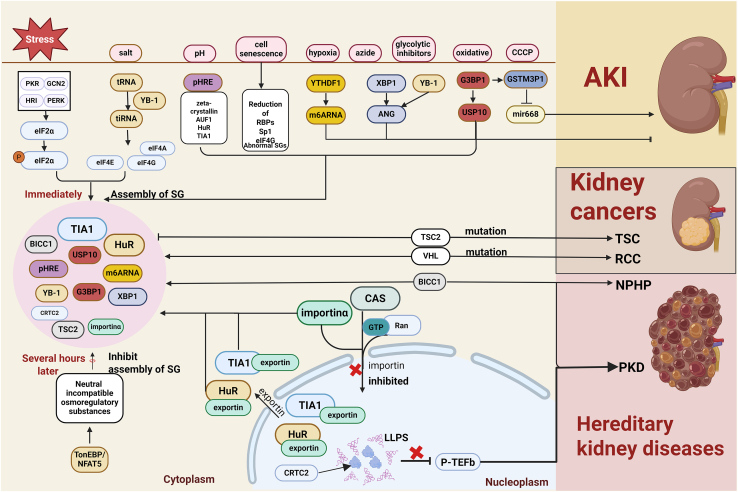
The relationship between kidney and SGs The figure mainly includes the cytoplasm and nucleus parts, as well as some signaling pathways that affect the formation of stress granules in the cytoplasm, which are closely related to the pathological state of the kidney and the occurrence of diseases.

Nephronophthisis (NPHP) is a rare autosomal recessive tubulointerstitial nephropathy. Associated ciliary abnormalities, cyst formation, and laterality defects are the most common genetic causes of end-stage renal disease.^238^^,^^239^ NPH family members have the function of binding RNA, and it has been previously shown that NPH family members can bind to known RBPs (such as Bicaudal-C1). Bicaudal-C1(BICC1) is associated with TIA-1-positive SGs forming in stress-induced translational inhibition responses, and is involved in maintaining tissue and organ integrity.^240^^,^^241^ Interestingly, BICC1 is also a target of chronic kidney diseases (CKDs), and its variation is associated with significant differences in longitudinal eGFR slope.^242^ Activation of the TOR kinase pathway was found in BICC1 mutant flies, another common feature of polycystic kidney disease (PKD).^243^ In addition, it was found that OFD1 (a centrosome/matrix protein) and Bicc1 cooperate to participate in the occurrence and development of renal cystic diseases in mammalian cells. OFD1 functionally controls the protein synthesis mechanisms of centrosomes, interacts with components of the pre-translational initiation complex (PIC) and eIF4F complexes, and regulates the translation of specific mRNAs in the kidney.^244^ Also, a recent study certifies that cAMP-induced nuclear condensation of CRTC2 (CREB-regulated transcription coactivator 2) promotes transcription elongation and cystogenesis in autosomal dominant PKD.^245^ The Figure 2 presented the complex role of stress in regulating RBPs within SGs, illustrating the diverse functions of SGs in kidney pathologic state in kidney cells and kidney diseases.

### Conclusion

A growing body of data suggests that SGs are functionally involved in the pathophysiology of kidney diseases through multiple pathways, including regulation of homeostasis at the RNA and protein levels. In the physiological diuretic and antidiuretic processes of the kidney, as well as pathological hypernatremia, metabolic acidosis, and aging, these chronic stresses lead to the continuous formation of SGs, which may be nephrotoxic. It is worth noting that in addition to various external stimuli, the aggregation of mRNA is closely related to the assembly and disassembly process of SGs.^9^ Disease-related RBPs mutations can also act as stressors; they accelerate the induction of abnormal SGs formation, and these pathological protein aggregates promote the occurrence and development of kidney diseases.^102^ Because of the specificity of cell types and stressors, SGs have different effects on the pathogenesis of each kidney diseases. We hypothesize that therapies targeting SG-mediated RNA metabolism in stressful environments may also improve the progression of kidney diseases. However, further exploration is needed to understand the behavior and role of SGs in the kidney in the context of renal pathogenesis. Particularly in nephrology, three fundamental questions demand urgent resolution: (1) The spatiotemporal coupling between SGs phase transitions and tubular epithelial cell injury-repair programs; (2) inter-organelle communication patterns governing SG-mediated stress adaptation; and (3) microenvironmental regulation of SGs dynamics within fibrotic niches. Establishing multiscale investigative single-molecule tracking,^14^^,^^246^ and spatial transcriptomics^101^ will be essential to decode the hierarchical control logic of phase separation in renal pathophysiology.

### Limitations of the study

There is relatively little research on SGs in the kidney, and the summary presented in this review has certain limitations. Due to space constraints, we apologize for any important literature in this field that may have been overlooked.

## Acknowledgments

This study was funded in part by grants from the 10.13039/501100001809National Natural Science Foundation of China (82170773).

## Author contributions

Conceptualization: J.C.Z. and H.S.; writing – original draft preparation and editing: J.C.Z.; supervision: H.S.; funding acquisition: H.S. Both authors read and approved the final manuscript.

## Declaration of interests

The authors declare no competing interests.
